# Editorial: The Embodied Brain: Computational Mechanisms of Integrated Sensorimotor Interactions With a Dynamic Environment

**DOI:** 10.3389/fncom.2020.00053

**Published:** 2020-06-18

**Authors:** Mario Senden, Judith Peters, Florian Röhrbein, Gustavo Deco, Rainer Goebel

**Affiliations:** ^1^Department of Cognitive Neuroscience, Faculty of Psychology and Neuroscience, Maastricht University, Maastricht, Netherlands; ^2^Maastricht Brain Imaging Center (M-BIC), Maastricht University, Maastricht, Netherlands; ^3^Department of Vision and Cognition, Netherlands Institute for Neuroscience, Royal Netherlands Academy of Arts and Sciences (KNAW), Amsterdam, Netherlands; ^4^Institut für Informatik VI, Technische Universität München, Munich, Germany; ^5^Center for Brain and Cognition, Computational Neuroscience Group, Department of Information and Communication Technologies, Universitat Pompeu Fabra, Barcelona, Spain; ^6^Institució Catalana de la Recerca i Estudis Avançats (ICREA), Universitat Pompeu Fabra, Barcelona, Spain

**Keywords:** sensorimotor integration, embodiment, neurorobotics, motor control, reinforcement learning and plasticity, neural computation

The paradigm shift toward an action-oriented view (Engel et al., 2013) stresses that cognition permits meaningful interactions with a dynamic environment and cannot be reduced to thinking-related mental representations. Consequently, the emerging field of embodied neuroscience has been inspired by recent achievements in robotics. At the same time, the fields of robotics and artificial intelligence increasingly turn to neuroscience to utilize insights on the neural underpinnings of sensorimotor interactions and embodied cognition.

As contribution to this integration of computational neuroscience, artificial intelligence, robotics and neurobiology, this Research Topic provides an overview of recent advances in sensorimotor integration and embodied cognition from a multidisciplinary perspective. A total of nine contributions present important scientific insights into embodied sensorimotor systems while another four contributions present comprehensive frameworks and toolchains that support the interdisciplinary study of embodied agents.

## Embodied Sensorimotor Systems

Embodied agents need to be able to autonomously and adaptively interact with their environment. Grossberg presents a large-scale visuomotor architecture: the Self-Organizing, Vision, Expectation, Recognition, Emotion, Intelligent, Goal-oriented Navigation model (SOVEREIGN; Gnadt and Grossberg, 2008). This architecture consists of several sensory, motor and memory components and is able to perform motor sequences under different motivational states as well as to learn more efficient sequences in response to rewards. Grossberg reviews the SOVEREIGN architecture as well as advancements in the field over the past decade and presents an updated version of the architecture, SOVEREIGN2. SOVEREIGN2 incorporates resonant dynamics which allow new perceptual, cognitive and navigational properties to emerge.

One highly complex cognitive aspect of sensorimotor integration, involving the recruitment and concerted interplay among a large number of cortical and subcortical brain regions, is action selection. Koprinkova-Hristova et al. capture this complexity with a biologically plausible large-scale architecture able to generate eye movement decisions. This architecture, implemented as a hierarchical spiking neural network (SNN), consists of multiple layers including the retina, several thalamic nuclei as well as cortical regions along the dorsal stream from V1 to the lateral intraparietal cortex. When probed with stimuli mimicking optic flow patterns of forward self-motion, the model selects eye movements that correctly align its gaze with the direction of self-motion.

Tekülve et al. approach a sequential pointing task from the perspective of dynamic field theory (Schöner and Spencer, 2016). Their contribution presents a spiking neural network (SNN) architecture comprised of: a perceptual subnetwork able to create a working memory representation of the visual scene, a motor subnetwork able to generate movement commands for a robotic arm, and a cognitive subnetwork able to represent positions in a sequence as well as to initiate shifts between positions. This architecture allows a robot to memorize a sequence of distinct objects (presented by a human), and subsequently point at these objects for random spatial arrangements of these objects.

Another robotic agent able to perform pointing movements is presented by Tieck et al.. They developed an SNN of the primary motor cortex that is able to adaptively combine motor primitives, a low-dimensional vocabulary of motor actions (Rizzolatti et al., 1988; Santello et al., 1998; Ciocarlie et al., 2007). A humanoid robot, utilizing this network, could successfully point at different targets marked on a plane.

The cerebellum is a key structure for sensorimotor control, as it coordinates voluntary movements through prediction and sensory feedback (Johansson and Westling, 1988; Wolpert and Flanagan, 2001; Xu-Wilson et al., 2009; Manto et al., 2012). Capolei et al. present a cerebellar microcircuit which, supplanted with a classic control method, allows for adaptive and robust control of a robot's movements as it balances a board with a rolling ball. The contributors show that cerebellar plasticity contributes to learning of dynamics related to arm-object interactions, and thus supports adaptive corrections to executed actions.

Inspired by the fact that evolution does not act on static, but rather on plastic systems learning from experiences in their environment, Massi et al. combine cerebellar plasticity with an evolutionary algorithm for optimizing quadruped robotic locomotion. Their control structure consists of a spinal central pattern generator (CPG) and a cerebellar adaptive controller able to learn online from feedback, while the parameters of the CPG are optimized offline via an evolutionary algorithm. Their results show that locomotion in a quadruped robot improves when the cerebellar controller is allowed to learn during evolutionary optimization as opposed to only afterwards. This suggests that parameters controlling the CPG need to be selected to benefit optimally from the adaptive controller.

The benefits conveyed by the cerebellum are intricately linked to its complex electroresponsive dynamics afforded by the plethora of cerebellar neuron types. Geminiani, Casellato et al. present a novel point neuron model able to capture the dynamics of several neurons of the olivocerebellar circuit. Their Extended-Generalized Leaky Integrate-And-Fire (E-GLIF) neuron is optimized to capture the input-output relationships of Golgi cells, granule cells, Purkinje cells, molecular layer interneurons, deep cerebellar nuclei cells and inferior olivary cells. Geminiani, Pedrocchi, et al. utilize the E-GLIF to investigate how single neuron dynamics in conjunction with geometrical modular connectivity profiles shape the dynamics exhibited by cerebellar circuits involved in eye blink classical conditioning. Their simulations produce response properties in Purkinje and deep nuclei cells similar to those reported *in vivo* when relying on the E-GLIF neuron model, but not when using simplified point neuron models.

This highlights the significance of neuron dynamics. Importantly, these dynamics are not only affected by neuron morphology. Vergara et al. argue that the balance between energy income, expenditure and availability determine neural dynamics to a significant extent. Importantly, the contributors argue, the effects of these factors manifest themselves at all levels from molecular to behavioral. In arguing their case, the contributors provide a comprehensive overview of energy demands of neurons culminating in the proposal of the Energy Homeostasis Principle.

## Toolchains and Frameworks

Constructing state-of-the-art embodied systems that are able to intelligently interact with their environment in a closed loop, requires the development of large-scale architectures incorporating several structural as well as functional components. The immensity of this task requires a high degree of collaboration among research disciplines. In order to facilitate such collaboration, universally available platforms, toolchains, and shared frameworks are indispensable.

One platform aiming to facilitate integration of several structural and functional components into an embodied agent is the neurorobotics platform (NRP; Falotico et al., 2017). Bornet et al. show how the NRP enables to connect models of diverse visual functions, developed by different research groups, into a coherent architecture. Their architecture, consisting of a retina model, a saliency model and a segmentation model, is able to explain visual crowding phenomena.

Jordan et al. present a novel toolchain for reinforcement learning in autonomous agents controlled by biologically plausible neural networks. This toolchain connects benchmarking tools from machine learning with network simulators from computational neuroscience. The collaborators demonstrate the functionality of the toolchain by implementing a rate neuron actor critic architecture in the NEST simulator (Gewaltig and Diesmann, 2007) and training on the grid world and mountain car environments.

The possibility to perform online reward-based learning with spiking neurons in the NEST simulator is provided by the Synaptic Plasticity with Online Reinforcement learning (SPORE) framework (Kappel et al., 2015, 2017, 2018). Kaiser et al. utilize the NRP to evaluate SPORE for training robotic agents on a closed loop reaching and lane-following task. The contributors show that SPORE was capable of learning shallow feedforward policies online for moderately difficult embodied tasks.

Mascaro et al. present an iterative loop between experiment and model simulation to refine and validate models with experimental data as well as adjust experiments based on simulations. The contributors demonstrate the feasibility of their iterative loop for two separate scenarios. In the first, the iterative loop allowed them to replicate the evolution of functional connectivity in the mouse brain after stroke using neural mass model simulations. In the second, the contributors integrated their iterative loop with the NRP to embody a spinal cord model of the mouse and were able to reproduce goal-directed forelimb movements. Such a framework that simulates all relevant components of an experimental study, facilitates the continuous integration of novel experimental results into model simulations. In turn, modeling results can contribute to ongoing improvements in experimental design.

## Conclusion

Understanding how an embodied brain can meaningfully interact with its dynamic external environment while managing inner homeostatic requirements is a challenging task. Indeed, identifying the functional capacities that an embodied nervous system needs to implement, the physical constraints it is subjected to as well as specifying representations, transformations and dynamics realizing these capacities requires input from computational neuroscientists, roboticists, machine learning experts, and neurobiologists. Contributions to this Research Topic reflect current advances in embodied action mechanisms across fields. However, for a comprehensive understanding of embodied cognition and its utilization in neurorobotics, it is essential that efforts become increasingly collaborative in the future. For this collaboration to be fruitful, support by an infrastructure enabling researchers to effectively integrate their empirical results and modeling efforts into large-scale closed-loop architectures will be indispensable. The frameworks and toolchains presented within the present Research Topic are an important step in that direction.

## Author Contributions

All authors acted as guest editors on the Research Topic. MS and JP wrote the Editorial.

## Conflict of Interest

The authors declare that the research was conducted in the absence of any commercial or financial relationships that could be construed as a potential conflict of interest.
